# The Latest Achievements in the Construction of Influenza Virus Detection Aptasensors

**DOI:** 10.3390/v12121365

**Published:** 2020-11-30

**Authors:** Ewelina Wędrowska, Tomasz Wandtke, Elżbieta Piskorska, Piotr Kopiński

**Affiliations:** 1 Department of Lung Diseases, Neoplasms and Tuberculosis, Faculty of Medicine, Nicolaus Copernicus University in Toruń, 85-094 Bydgoszcz, Poland; tomasz_wandtke@wp.pl; 2 Department of Pathobiochemistry and Clinical Chemistry, Faculty of Pharmacy, Nicolaus Copernicus University Collegium Medicum, 85-094 Bydgoszcz, Poland; piskorska_e@cm.umk.pl or

**Keywords:** aptasensors, aptamers, influenza, flu, virus

## Abstract

Aptamers are short fragments of nucleic acids, DNA or RNA that have the ability to bind selected proteins with high specificity and affinity. These properties allow them to be used as an element of biosensors for the detection of specific proteins, including viral ones, which makes it possible to design valuable diagnostic tools. The influenza virus causes a huge number of human and animal deaths worldwide every year, and contributes to remarkable economic losses. In addition, in 2020, a new threat appeared—the SARS-Cov-2 pandemic. Both disease entities, especially in the initial stage of infection, are almost identical in terms of signs and symptoms. Therefore, a diagnostic solution is needed that will allow distinguishing between both pathogens, with high sensitivity and specificity; it should be cheap, quick and possible to use in the field, for example, in a doctor’s office. All the mentioned properties are met by aptasensors in which the detection elements are specific aptamers. We present here the latest developments in the construction of various types of aptasensors for the detection of influenza virus. Aptasensor operation is based on the measurement of changes in electric impedance, fluorescence or electric signal (impedimetric, fluorescence and electrochemical aptasensors, respectively); it allows both qualitative and quantitative determinations. The particularly high advancement for detecting of influenza virus concerns impedimetric aptasensors.

## 1. Introduction

There are many viruses in the environment that infect humans, other animals or both, around it. Some of them contribute to the development of diseases. The most pathogenic viral strains can cause diseases leading to permanent damage to health and even death. However, they always contribute to economic losses [1,2]. In order to avoid the undesirable consequences of either viral, health and economic infections, not only effective therapeutic solutions are necessary, but most of all, reliable, effective and quick diagnostic methods with high sensitivity and specificity, which, as a result of the diagnosis, will allow for either appropriate treatment, quarantine or both [3,4,5].

There are many viruses that are extremely dangerous to humans. Among them, one of the most important is the influenza virus, which not only easily mutates, causing further epidemics in humans and animals, but some of its strains also cause infections with low survival rates. A particularly important strain in this context is the H5N1 avian influenza virus, which causes severe economic losses and causes death in farmed poultry. Moreover, under favorable circumstances it also infects people, causing an infection characterized by high, as much as 60% mortality [6,7,8]. A classic example of the lethal human influenza virus potential was the Spanish flu, which decimated the world’s population between 1918 and 1920, causing over 50 million deaths worldwide [9]. There are also many other dangerous viruses, such as HBV or HCV. They constitute a serious health problem contributing to liver dysfunction and even the development of hepatocellular carcinoma in humans [10,11]. There are also deadly viruses such as Ebola for which there is still no effective treatment. At the turn of 2013/2014, the virus spread widely through African countries. There are serious and well-founded concerns about its expansion to other geographic regions [12]. Apart from those mentioned, there are many other viruses, such as HIV, Zika, et cetera, which also constitute a serious diagnostic and therapeutic problem [13,14]. The year 2020 also brought a whole new challenge in the form of the SARS-CoV-2 virus that causes COVID-19. This is an extremely unfortunate event because the death rate caused by the virus is relatively high and the virus itself is highly contagious. An extremely unfavorable coincidence is the fact that initial symptoms of SARS-CoV-2 infection are deceptively similar to infection with the group virus.

It seems that the key to efficiently dealing with the expansion of viruses dangerous for humans is a quick and correct diagnosis, often also involving the differentiation of infections with a similar course as in the case of the influenza virus and SARS-CoV-2. Among the standard diagnostic methods used in the detection of viral infections, enzyme-linked immunosorbent assay (ELISA) and molecular methods based on the polymerization chain reaction dominate [15,16,17]. The first have a major disadvantage—they allow detection of infection at the time of developing immunity, in other words, antibody production. This process usually takes place a few days or weeks after infection, which makes it impossible to make a quick and reliable diagnosis right after infection, and additionally prevents the application of immediate pharmacological prevention. The second group of diagnostic methods, based on the polymerization chain reaction, for the detection of viral nucleic acid is very sensitive and allows the detection of infection in a period incomparably shorter than that of immunoenzymatic methods. However, their use is extremely costly and therefore not routinely used. Moreover, in the case of both types of methods, their use is time-consuming [15,16,17,18,19]. Aptamers seem to be an alternative to the above-mentioned problems of virological diagnostics, the use of which guarantees high sensitivity and specificity of detection, and is also relatively cheap. The use of aptamers as components of virological sensors is a promising panacea for the problems of currently used diagnostic solutions [15,20,21].

Aptamers are short (usually no longer than 90 nucleotides) fragments of nucleic acids, DNA or RNA that bind selected proteins (as well as other chemical molecules) in a specific manner and with high affinity. They show a number of advantages over monoclonal antibodies: they are stable at room temperature, homogeneous, easy to modify and, above all, simple and cheap to obtain [8,22]. Aptamer molecules are obtained by the SELEX method (systematic evolution of ligands by exponential enrichment) developed and used for the first time by the Tuerk and Gold teams [23] and Elington and Szostak in 1990 [24]. Briefly, this technique relies on repeated cycles of selection and replication of a combinatorial library of DNA or RNA molecules of a specified length in vitro under selected conditions by the recipient. Since the first application of this method, many variations of this technique have been developed that either accelerate the aptamer production, enhance or both; either the specificity, affinity or both; of these molecules for target molecules. The process of obtaining aptamers, as well as their more in-depth characteristics, is not the subject of this study, therefore, in order to find out more about these issues, we encourage you to read our other article, where these issues are presented in detail [8]. Binding of target proteins with high specificity and affinity by aptamers often results in their function being blocked. Such action can be of great importance in the treatment of various diseases, including viral ones. Much research has already been carried out in this area. However, the high specificity and affinity of the binding of target molecules by aptamers have made them attractive not only for therapeutic but also diagnostic applications, where quick and reliable diagnosis in the case of viral diseases is often crucial and may favor either the prompt treatment of the patient (if therapy exists), its isolation or both, which in turn prevents further spread of the disease [3,4,5,25].

The use of aptamers in the diagnosis and therapy of diseases brings great benefits and is the subject of many research projects. The authors of extensive review papers compare their properties in comparison with monoclonal antibodies, probably due to the fact that they are a direct competition for this diagnostic tool. However, this technique is not without disadvantages, although they remain in significant disproportion compared to the advantages. The most important of them are listed in Table 1.

In our previous article, “Aptamers in diagnostics and treatment of viral infection” published in 2015 in the journal “Viruses”, we presented in detail the current research and clinical achievements regarding the diagnosis and therapy of viral diseases with the use of aptamers. Since then, there have been many new and noteworthy reports on virological diagnostics. When creating this study, we decided to review these solutions with particular attention to the influenza virus, as in our opinion, research into the diagnosis of this virus seems to be the most advanced. In addition, due to the current epidemiological threat caused by the SARS-CoV-2 virus, the infection of which resembles the course of an influenza virus infection, the solutions for the detection of the influenza virus developed so far may be of great importance in the differentiation of both infections. This is even more significant in that research into the use of aptamers in the detection of the SARS-CoV virus-2 requires time is just starting. The current work is a kind of update of the content of the article mentioned above—it focuses on the current reports on the use of aptamers in the diagnosis of the influenza virus.

## 2. Flu Virus Diagnosis

The influenza virus, along with SARS-CoV-2 infection, represents one of the most challenging challenges in virology today. It is an enveloped virus [31]. Its molecules contain seven (influenza type C and D) or eight (influenza type A and B) single-stranded negative-sense RNA segments that make up its genome. The genome encodes, among others, matrix protein M1 and viral nucleoproteins, which are used to distinguish its three main types—there are three types: A, B and C [31]. In turn, on the surface of the influenza particles there are two types of glycoproteins that have been encoded in the genome—hemagglutinin and neuraminidase. The differences in their structure have become the basis for distinguishing subtypes of influenza viruses [31,32]. Type A and B viruses can infect humans [31]. Type A viruses seem to pose the greatest threat to humans, mainly due to the phenomena of drift and genetic shift, which are easily affected [33,34]. This phenomenon most often concerns the viral hemagglutinin of the virus—a protein involved in the process of infection of host cells (attaching to their surface and penetrating into them). Neuraminidase is involved in the transmission of the virus. In nature, there are 15 types of hemagglutinin and 9 types of neuraminidase, which are the basis for the differentiation of influenza A virus type [32]. The variability resulting from the phenomena of drift and genetic shift is the main cause of difficulties in the development of vaccines and drugs against this type of virus. These phenomena also affect the design of diagnostic solutions, which they do not facilitate either [33,34].

A particularly important case, which receives a lot of attention and research, is the avian influenza virus—H5N1 [35,36], that belongs to the group of influenza type A viruses. Until 2012, according to FAO findings, it caused epidemics that resulted in nearly 400 million deaths among farmed poultry, which contributed to significant economic losses (over USD 20 trillion) for farmers around the world [37]. The first cases were found in 1990 in Southeast Asia and later also in the Middle East and Europe [35]. However, what is extremely worrying, cases were also reported in sixteen countries around the world, among people [38]. Their occurrence is favored, in the case of the avian influenza virus, by the high frequency of mutations and the ease of reassortment of this strain with other strains of the influenza virus [35]. Infection with influenza type A virus causes infections of the upper respiratory system, which are manifested by fever, nasal congestion and headache [39,40]. Between years 2003 and 2016, 844 cases of infection with this strain in humans were detected, according to a report by the World Health Organization. They were characterized by a very high, as much as 53% mortality (449 deaths) [38]. In 2013, the first case of human infection with the H7N9 strain of avian influenza was also confirmed, which, like the H5N1 strain, is characterized by high mortality in humans, reaching over 36% [41,42]. The strains of influenza type A virus—H1N1 and H3N2—are also the most infectious strains of this virus for humans and can cause large and rapidly spreading epidemics [43,44].

B influenza type viruses only infect humans and seals. The ailments they cause are usually much less severe than those caused by infection with the group of type A viruses [45]. The severity of symptoms caused by infection with C influenza type is even less.

The threat and economic losses associated with the outbreak of a potential epidemic prompted us to undertake further research aimed at developing not only therapeutic measures, but most of all, diagnostic solutions that will ensure the possibility of immediate, on the spot, detection of the virus and taking immediate preventive actions. A significant drawback of the currently used solutions is the long waiting time for the result, the release of which is preceded by a relatively long time necessary to perform analysis in laboratory conditions [34,35,46], while it has been proven that the initiation of antiviral treatment within 24 h (e.g., Tamiflu preparation) from the onset of symptoms significantly reduces the duration of the infection and its severity. This fact significantly influences the activities of scientists whose aim is to accelerate the diagnostic process, and thus reduce the negative effects of infection with influenza viruses [40,47]. The most frequently used analyses are three groups of methods: viral cultures for the multiplication of the virus and its subsequent identification, genetic methods consisting of the detection of the viral genome and immunological methods consisting of the detection of antiviral antibodies or virus antigens. The first group of methods includes RT-PCR and real-time polymerase chain reaction (PCR), and the second group of ELISA tests or rapid influenza diagnostic test (RITD). The use of the former is very time-consuming and expensive—analysis cannot be performed on site; the test material must be collected from the patient and sent to the laboratory, which extends the identification time and postpones the initiation of treatment (e.g., the anti-influenza preparation Tamiflu should be administered within 24–48 h from the onset of symptoms, which in the case of using this type of diagnostic methods becomes impossible) [48]. The second group of methods, in turn, is often characterized by insufficient sensitivity and specificity (cross-reactions against other viruses and pathogens were observed during detection due to the production of insufficiently specific antibodies) [49] compared to molecular methods. For example, RITD tests have extremely low diagnostic sensitivity (10–70%), resulting in a high number of false negative results. In the case of classic ELISA tests, they more often rely on the detection of antibodies than the virus itself [33,35,46,50,51,52,53,54]. Methods based on the culture of cells, most often PMK (primary rhesus monkey cell line) and MDCK (Madin–Darby canine kidney cell line) infected with the virus isolated from patients, favor its multiplication and effective and easier identification on the basis of viral antigens present in numerous cultures. However, this method is extremely time-consuming and labor-intensive. It takes 3–14 d to determine the type of virus [55].

Biosensors are an interesting alternative to the diagnostic methods currently used to detect viruses. These are devices that use a biological element capable of specifically capturing a viral particle, for example, it may be an aptamer. Most often, it is combined with a processing unit which, due to the binding of the target molecule by a specific aptamer, emits a signal depending on the type of biosensor, which is synonymous with the detection of a specific pathogen [35]. In our previous work [8], we reported that at least several research teams had developed aptasensors to detect influenza viruses, including avian influenza. They used, inter alia, the surface plasmon resonance method [56,57,58,59,60], quartz crystal microbalance [61,62,63,64], the phenomenon of fluorescence [65,66] as well as electrochemical processes [67,68,69,70,71]. At that time, the proposed solutions were characterized by higher sensitivity and specificity than the constructed immunosensors [36], but they did not meet the requirements for solutions that could be used “in the field”. First of all, the use of aptasensors was relatively time-consuming and cost-intensive, and was characterized by a rather complicated methodology. For example, using a sensor based on the SPR technique required about 90 min to obtain results, while a waiting time of no more than 30 min is expected for fast detection methods. It seems that a lot has changed in this regard over the last five years, so we again took information on the latest advances in virological diagnostics using aptasensors, which are presented later in this paper.

One of the molecules most frequently targeted for aptamer detection in influenza virus is hemagglutinin. It is a protein that plays a key role in the process of infection of host cells, as well as in the formation of immunity. It is known that the production of antiviral antibodies mainly involves the production of molecules directed against this particular viral protein. It occurs on the surface of the virus as a trimeric spike [64].

## 3. Impedimetric Aptasensors

One of the most frequently used and developed biosensors in the diagnosis of influenza virus, including the avian subtype H5N1 (but not only), are impedimetric detectors. This is due to the possibility of their miniaturization and low production costs, which is always an important factor determining the development of a given technology. Their operation is based on electrochemical changes occurring in the tested material, the occurrence and subsequent detection of which allows confirming or excluding the presence of the molecules sought. First of all, impedimetric techniques measure the actual changes of impedance or resistance that occur when a target molecule binds to a biosensor. The methods of detecting these changes depend on marking elements (direct or indirect labeling) or can be label-free. In addition, it has been proven that the use of selected materials such as, for example, gold nanoparticles, carbon nanotubes and others, in the construction of impedimetric aptasensors or their elements (most often microelectrodes included in biosensor) multiplies detected signal changes, which increase the sensitivity of the test. Moreover, additional advantages resulting from the combination of an impedimetric biosensors with different types of microelectrodes are: the fast kinetics of the reaction, shorter detection time resulting from the fast sensor response and what was mentioned earlier—the amplification of the emitted signal in relation to the background signal, which allows for minimizing the volume of the tested sample [35,72,73]. In addition, the use of a connection often minimizes the amount of preparatory steps needed to be performed with the test material. This improves the psychological comfort of a diagnostician’s work and reduces the risk of infection resulting from more frequent contact with the tested material [73]. Aptamers have proven to be effective detectors of viral particles that could be used in virological biosensors. Their use is associated with a very low background signal, high repeatability of results and high sensitivity of the device [33,35]. For most solutions, three times the maximum standard deviation of the measurement made for the control sample, which is the so-called background signal, was adopted as the standard for the lower detection limit [36,74].

Lum et al. developed an impedance biosensor containing a DNA aptamer capable of detecting H5N1 avian influenza virus particles. The described sensor uses a gold electrode placed in a microflow chamber, which is coated with streptavidin in such a way that it can interact and bind the detection element on its surface—a biotinylated aptamer specific for the H5N1 virus. The presence of the virus in the tested material was confirmed by the increase in the impedance magnitude, which was directly proportional to the virus concentration in the tested material and resulted from the blockage of the ion flow caused by the binding of the virus by a specific aptamer coated on the microelectrode. The biosensor allowed for selective detection of H5N1 avian influenza virus. The presence of other types of influenza virus in tested material, such as H1N1, H2N2 and H7N2 did not interfere with the detection process. It was a direct evidence for high specificity of the constructed biosensor. The high specificity of the sensor resulted not only from the presence of the aptamer, but also from the lack of the need to perform the blocking procedure. As a consequence, when aptamers are used, the background noise is significantly reduced, which increases the specificity of biosensors. Moreover, the biosensor constructed by Lum et al. was also characterized by high sensitivity. The attainable detection limit was not lower than in previous solutions constructed by the same [75] and other research teams [64]—the sensor allowed detection of the virus in the amount of 0.0128 hemagglutinin units (HAU). Hemagglutination (HA) stands for agglutination of red blood cells induced by the presence of virus. One HA unit (HAU) is the amount of virus needed to agglutinate an equal volume of a standardized RBC suspension, or one HAU in a virus suspension is measured by the amount of virus dilutions made equal to the amount of HA titers [35]. Use of described biosensor for virus detection was fast (only 30 min). This was due to elimination of the need to mark the tested material and also elimination of the necessity of its condensation. Moreover, for the first time, the time, sensitivity and, above all, the form of the biosensor achieved, enabled its possible use in the field. Previously, the proposed solutions [64] with similar time parameters and detection sensitivity were not adapted to use outside the laboratory [76]. The aptasensor designed by Lum et al. was miniature, had low energy requirements and was easy to use [35].

An aptasensor constructed in an analogous way (biotinylated DNA aptamer [64], streptavidin-coated gold microelectrode, impedance measurement) also using a similar method of operation as that designed by Lum et al. [35], was edited by Karash et al. [36]. It was used to detect the same virus strain—H5N1—whose particles were inactivated. The aptamer used was characterized by high virus binding affinity; the dissociation constant was 4.65 nM. One of the basic differences in the approach was the use of the microelectrode blocking step during the analysis, which was performed with the use of polyethylene glycol, and the use of a signal amplifier—thiocyanuric acid, which created a kind of cross-linking binding gold nanoparticle covalently linked with aptamers, which increased the generated impedance signal at least 48 times [36]. Although the solution proposed by Karash et al. was slightly less sensitive and longer in execution than that proposed by Lum et al., the limit and time of detection were satisfactory [35,36]. The detection limit for the discussed aptasensor was 0.25 HAU for the purified virus and 1 HAU for the virus introduced into swabs collected from the trachea of breeding poultry. The first of the mentioned detection thresholds is lower than in the case of the conventional RT-PCR test (0.5 HAU) and higher than the detection threshold of the real time PCR method (possible detection of 0.125 HAU of the virus). On the other hand, the performance of the real time PCR test is significantly more expensive, longer and, above all, must be performed in a laboratory. In the case of the described aptasensor, the detection can take place on site, in the field. On the other hand, the specificity of the aptasensor was confirmed in the presence of influenza virus strains other than H5N1, which generated only a minimal impedance signal. Even 90% similarity of the hemagglutinin sequence of the H5N2 avian influenza virus strain did not cause it to bind to the aptamer, which proved its high specificity for the H5N1 strain. The detection time was 45 min when using the variant without signal booster. A sample with a volume of only 30 microliters was enough to carry out the reaction, which is an unquestionable advantage in the case of having very small samples. Moreover, Karash et al. made a concise economic analysis for the proposed solution, stating that the total cost of making a single sensor, excluding the cost of the personnel performing the test, is only $13, of which the main cost is the microelectrode constituting the core of the aptasensor. The authors of the study speculate that mass production would reduce the total cost of one test to as low as $3 [36]. Moreover, the construction of aptasensors based on the solution proposed by the authors of the publication was cheaper than in the cases postulated by other authors. For example, the generation of an aptasensor proposed by Wang et al. using quartz crystal electrode generated a cost of nearly $20/test [64], and the production of microfluidic chips proposed by Lum et al. [35] cost as much as $100/chip. The cost-quality ratio of the compared tests (their sensitivity, specificity and execution time) seems to be in favor of the solution proposed by Karash et al. [35,36,64].

A solution based on the detection of the avian influenza virus using an electrochemical impedance spectroscopy using an aptasensor was also proposed by Kirkegaard et al. Their main goal was to reduce the detection time and sensor production costs. For this purpose, instead of conventional metallic electrodes, they used an electrode made of PEDOT:PSS (poly (3,4-ethylenedioxythiophene) polystyrene sulfonate) [39]. Such electrodes are much easier to manufacture than conventional metal electrodes. Moreover, the cost of materials necessary for their production is significantly lower, and their stability and conductivity are sometimes higher than in the case of traditional electrodes [77]. The primary biological component responsible for the capture of H1N1, H3N2 and H2N2 avian influenza particles was the hemagglutinin-specific single-stranded A22 DNA aptamer of the virus, which had already been selected by the team of Jeon et al. [78], and finally it was modified by introducing TC-tag into its structure, which made it possible to immobilize it on the surface of the created electrode. According to the authors of the study, the developed aptasensor enabled detection in no more than 30 min [39]. The goals set by the team were achieved, although there is no information in the publication about the sensitivity of the developed solution.

Bai et al. developed sensors to detect a different strain of influenza A, H1N1. For its construction, a DNA aptamer was used, which was selected against inactivated whole particles of this virus. Typically, this approach produces aptamers that are specific for conserved regions of viral antigens. The authors developed two diagnostic tests. The first—the ELONA test—was the equivalent of the ELISA test, in which monoclonal antibodies were replaced with molecules of a selected aptamer. This allowed obtaining the H1N1 detection limit (LOD) defined as the signal-to-noise ratio value, S/N = 3 at the level of 0.3 ng/uL. The second diagnostic device constructed with the use of the selected aptamer was the electrochemical impedance aptasensor, the use of which allowed achieving a sensitivity of 0.9 pg/uL—nearly 300 times lower than in the case of the designed ELONA test. The thiolated aptamer was immobilized on the surface of the gold microelectrode. The constructed aptasensor allowed for one-step detection without prior lysis of viral particles or extraction of their proteins. In addition, it showed significant specificity—it detected the H1N1 and H3N2 influenza virus strains. Interestingly, the research team observed that lower aptamer density on the electrode surface favored an increase in specificity, while its increase favored an increase in sensitivity at the expense of sensor specificity. The latter observation, in particular, may be important when analyzing samples with a very low content of viral particles—as in the case of, for example, throat swabs [33].

## 4. Fluorescent Aptasensors

An important type of aptasensor is also those based on the measurement of fluorescence. Many other diagnostic methods are based on the detection of this phenomenon. These methods are used routinely nowadays mainly due to the high sensitivity and ease of performing the procedure [34].

In recent years, Pang et al. have developed a solution that enables the detection of H5N1 avian influenza virus hemagglutinin in human plasma. For this purpose, they used Ag-SiO_2_ nanoparticles coated with an aptamer rich in guanine residues. Interestingly, this aptamer underwent conformational changes depending on whether there were molecules of the detected protein in its vicinity or not [34]. It has been proven many times that nucleic acids showing the II-order structure, including aptamers obtaining the G-quadraduplex structure, are able to bind various dyes and induce their fluorescence [79]. The team of Pang et al. used an aptamer with just such properties. It was able to obtain G-quadraduplex structures in the presence of viral hemagglutinin and consequently to bind Thiazole Orange—a dye that only emits fluorescence upon binding to a conformationally altered aptamer [80]. Importantly, any fluorescence-based diagnostic method must be strong enough. In the case of the solution proposed by Pang et al., the fluorescence signal was repeatedly amplified as a result of the use of Ag-SiO_2_ nanoparticles [34,66]. In the proposed solution, the background signal emitted by the free Thiazole Orange present in the reaction mixture was remarkably low. The viral protein was detected in human plasma in a one-step process, as early as 30 min and at a concentration of only 3.5 ng/mL (the detection limit for the aqueous protein solution was even lower, only 2 ng/mL). It was a solution that, due to the extremely short detection time, according to the authors, could be used to detect the virus directly at the site of sample collection. It was faster, more sensitive and more specific than enzyme immunoassays used for the same purpose [34].

In turn, Tseng et al. proposed a detection method specific for the influenza virus of the H1N1 type. In order to detect it, they used a microfluidic system with a sandwich-based system and fluorescence emission. The virus capture was possible, as in the previous cases, thanks to the incorporation of an aptamer into the system, characterized by very high specificity and binding affinity for virus particles. Two identical aptamers were used in the designed test—one detected the virus, the other increased the specificity of the reaction and was responsible for the emission of fluorescence since it was conjugated to the fluorescent marker. As in the solution proposed by Pang et al., this time the detection time was also very short and did not exceed 30 min, and the entire process was automated. The detection limit of the designed solution was only 0.032 HAU, which, according to the authors of the study, made the designed test comparable in terms of sensitivity with tests involving the use of monoclonal antibodies or PCR techniques [54] and approximately 30 times more sensitive than conventionally used serological solutions of the RIDT type [81]. What is more, the authors of the study pointed out that the use of aptamers brings many benefits, since it is known that they are more stable (in various temperature and humidity ranges), easier to store and cheaper to obtain (chemical synthesis) than monoclonal antibodies in standard enzyme immunoassays applications today [26,54].

Among the achievements in the field of fluorescent sensors is the aptasensor developed by Wang et al. The solution proposed by the authors made it possible to detect three different types of influenza virus, including two type A—H1N1, H3N2—and influenza B virus type.

As in the previously mentioned cases, the detection element was a fluorescently labeled DNA aptamer. It was integrated with a relatively small microfluidic system. This aptamer predictably changed its conformation depending on the type and concentration of ions present in the environment. These changes enabled the simultaneous detection of various types of influenza virus in no more than 20 min and with a detection limit of 3.2 HAU, which makes the described microfluidic system a potential tool for on-site diagnostics in the office of, for example, a doctor [40].

## 5. Electrochemical Aptasensors

The diagnosis of influenza virus is quite often based on the use of electrochemical immunosensors. The principle operation of this type of technique is based on a chemical reaction between the sensor and a chemical substance, during which an electric signal is generated with intensity proportional to the content of the tested factor. The speed of electrochemical analyses, as well as their high sensitivity, selectivity and simplicity in performing determinations, make them suitable for real-time virus detection [82]. Currently, there is a lot of research in this field, and below are selected projects that undoubtedly deserve attention.

The clinical need for rapid differentiation of influenza virus subtypes is directly related to their differential susceptibility/susceptibility to treatment. This mainly concerns the H1N1 and H3N2 subtypes that are widespread in humans. Accordingly, the team of Bhardwaj et al. in 2019 [83] presented the characteristics of two candidates for ssDNA aptamers for the selective detection of influenza AH1N1 virus (V46 and V57—dissociation constants were 19.2 nm and 29.6 nm, respectively), the action of which is based on targeting the recombinant mini-hemagglutinin protein (mini-HA—the stable stem region of HA) and whole H1N1 viruses. In the course of the conducted analysis, the authors observed that the V46 aptamer exhibits greater affinity for mini-HA and the H1N1 virus subtypes. For this reason, in a further stage of the research, it was incorporated into an indium tin oxide-based electrochemical sensor. The results of the analysis turned out to be very promising as the detection limit for the H1N1 virus was 3.7 plaque forming units per milliliter (PFU/mL), and its selectivity was confirmed by distinguishing six H1N1 virus strains from four different influenza A virus subtypes. Therefore, the authors’ suggestion is understandable that the use of the V46 aptamer in combination with an electrochemical sensor may find application in the rapid differentiation of the H1N1 subtype of the influenza virus, and possibly also may be useful in its neutralization, which will certainly find therapeutic application. Moreover, the detection of H1N1 virus using this aptasensor is faster than standard ELISA assays and nucleic acid based techniques.

Furthermore, Bhardwai’s team and colleagues constructed a vertical flow-based paper immunosensor for the rapid electrochemical and colorimetric detection of the H1N1 influenza virus with high sensitivity [84]. For this purpose, a sample pad with two pore sizes/DP was used—double pore size, a conjugate cartridge and a nitrocellulose membrane strip. The DP sample pad constructed in this way had the desired filtration properties, which was confirmed by using fluorescent beads, showing that only small-sized bio-particles such as viruses can pass through it. The measurements were performed using the electrochemical impedance spectroscopy (EIS) technique, where working, opposing and reference electrodes were used, which were, respectively, a gold paper electrode, carbon paste electrode and Ag/AgCl electrode. The thus constructed measuring set made it possible to detect the H1N1 virus at the concentration of 3.3 PFU/mL (PBS) and 4.7 PFU/mL (saliva). At the same time, the color signal emitted by the free HRP-Ab was assessed by means of a colorimetric scanner. Its use made it possible to detect the virus in the titers of 1.34 PFU/mL (PBS) and 2.27 PFU/mL (saliva). According to the authors, the simultaneous use of these two methods will help to reduce the number of false results, and the device for simultaneous qualitative and quantitative analysis will simplify and speed up the diagnosis of the influenza virus. 

The H1N1 influenza virus, due to its dangerous impact on human health, especially in children and the elderly, has become the subject of research by Kushawa et al. [85]. The authors, looking for quick diagnostic methods, were the first to apply the procedure of acquiring aptamers with the use of multiple targets SELCOS (systemic enrichment of ligands by competitive selection), also known as competitive non-SELEX, in which, unlike the techniques used so far, the polymerase chain reaction (PCR) is not used. The aptamers obtained in this way show the ability to detect targets with a very similar structure, which proves their extremely high selectivity. The aptamer obtained in this way was used as an element of the electrochemical diagnostics system (Apta-DEPSOR) allowing the selection of aptamer pools with high affinity (for H1N1 KD (dissociation constant) = 82 pM, for H3N2 KD = 88 pM) and low cross activity. This study used a single-use, three-electrode screen-printed (DEP) chip. This three-electrode electrochemical analysis system uses a carbon working electrode, a counter electrode and an Ag/AgCl reference electrode. The electrochemical detection was based on the Au NPs electro-oxidation reaction. An increase in DPV (differentia pulse voltammetry) signal was observed with an increase in Au NPs captured by the H1N1 protein on the surface of the working electrode. A reduction in the DPV signal was in turn observed when most of the H1N1 protein was captured by the aptamer strands in the solution. This aptasensor detected H1N1 in the dynamic range of 0.4–100 µg/mL, and the LOD was 0.51 µg/mL. This research represents a huge step towards the real transfer of the rapid and reliable diagnosis of the H1N1 virus to the field.

## 6. Conclusions

The diagnosis of viral diseases requires not only the use of sensitive and specific methods, but they must also be fast, cheap and widely available. An ideal diagnostic test should be possible in the doctor’s office and the result should be available in a short time. This is crucial in preventing the spread of epidemics of viral diseases such as the flu and COVID-19.

Aptamers are a specific, clean, cheap and quick solution with enormous potential in the diagnosis of viral diseases. Numerous studies summarized in this paper have demonstrated their effectiveness in detecting pathogenic strains causing influenza in humans and other animals. First of all, the progress of biological and technical sciences allowed for the miniaturization of the applied solutions, significantly simplified them and lowered the cost of their production and implementation. Even before 2015, the results of research work and the use of aptasensors in the diagnosis of diseases caused by viruses did not allow their use in the field, outside the laboratory. Currently, the solutions developed have mostly solved old problems. Among the aptasensors dedicated to the detection of the influenza virus, various solutions are used, including impedimetric, fluorescent and electrochemical. In particular, research and impedimetric aptasensors show, in our opinion, very advanced and the best relative results. Before their use, however, final studies confirming the effectiveness of their use are necessary. These studies should be performed as quickly as possible, since the potential of aptasensors in the diagnosis of viral diseases, including influenza, seems to be enormous, and the widespread COVID-19 epidemic, due to the similarity of symptoms of the influenza infection will require reliable diagnostic tools differentiating infections by influenza viruses and SARS-CoV-2, especially in the approaching period of increased flu cases in the northern hemisphere.

## Figures and Tables

**Table 1:** Main advantages and disadvantages of using aptamers in diagnostics [25,26,27,28,29,30].

Advantages	Disadvantages
- short duration of chemical synthesis (a few days) - relatively low cost of production - high stability of the obtained particles—no variability between batches - low immunogenicity - affinity and specificity similar to monoclonal antibodies - the possibility of increasing the affinity to the analyte by increasing the rounds of SELEX (systematic evolution of ligands by exponential enrichment) - high selectivity - the ability to selectively adjust to selected extracellular and intracellular targets (proteins, nucleotides, amino acids, small molecules, ions, viruses, living cells) - the possibility of their immobilization on many surfaces and coupling with nanoparticles and drugs - small particle size compared to monoclonal antibodies ensures higher sensitivity and lowers the limits of detection of analytes - ability to regenerate and re-use (renaturation after denaturation allows rebinding of the analyte) - possibility of chemical modification without loss of binding capacity or performance - possibility of adaptation to operation in non-standard temperatures, solvents, buffers	- aptamer sequence determination using the SELEX technique is long-lasting (several months) - SELEX carries extremely high costs - small size contributes to a high rate of excretion from the body - unmodified aptamers are susceptible to degradation by serum nucleases
